# Reference values for amino acids and acylcarnitines in peripheral blood in Quarter horses and American Miniature horses

**DOI:** 10.1186/s13028-015-0144-9

**Published:** 2015-09-29

**Authors:** Irám Pablo Rodríguez-Sánchez, Víctor Manuel Treviño-Alvarado, María del Rosario Torres-Sepúlveda, Liliana Aracely López-Saldaña, Gustavo Ponce-García, Graciela Areli López-Uriarte, María del Consuelo Ruiz-Herrera, Diana Elisa Zamora-Ávila, Jesús Zacarías Villarreal-Pérez, Guillermo Dávalos-Aranda, Laura Elia Martínez-de-Villarreal

**Affiliations:** Departamento de Genética, Hospital Universitario “Dr. José Eleuterio González”, Universidad Autónoma de Nuevo León, Monterrey, Nuevo León Mexico; Tecnológico de Monterrey Cátedra de Bioinformática, Campus Monterrey, Monterrey, Nuevo León Mexico; Departamento de Genética, Facultad de Medicina Veterinaria y Zootecnia, Universidad Autónoma de Nuevo León, Monterrey, Nuevo León Mexico; Departamento de Zoología de invertebrados, Facultad de Ciencias Biológicas, Universidad Autónoma de Nuevo León, Monterrey, Nuevo León Mexico; Servicio de Endocrinología, Hospital Universitario “Dr. José Eleuterio González”, Universidad Autónoma de Nuevo León, Monterrey, Nuevo León Mexico

**Keywords:** Amino acid, Acylcarnitine, Metabolic profile, Miniature horse, Quarter horse, Tandem mass spectrometry

## Abstract

**Background:**

Free amino acids and acylcarnitines circulating in the blood can be used for diagnosis for metabolic illness and imbalances. To date, the normal reference ranges of amino acids and acylcarnitines in horse peripheral blood have not been established. In this study, the concentrations of 12 amino acids and 26 acylcarnitines were determined by tandem mass spectrometry in complete blood from 100 healthy horses (50 Quarter horses (QH) [23 males and 27 females] and 50 American Miniature horses (AMH) [15 males and 35 females]) with no signs of metabolic disease. The means and standard deviations were determined and data statistically analyzed.

**Findings:**

Concentrations of short, medium, and long chain acylcarnitines were significantly higher in male AMH than in male QH. The concentrations of the amino acids alanine, arginine, glycine, proline (glycogenic), and leucine (ketogenic) were higher in the QH than in the AMH. Female AMH had higher concentrations of propionylcarnitine, leucine, proline, arginine, and ornithine than female QH.

**Conclusions:**

Normal reference ranges of amino acids and acylcarnitines were established for AMH and QH. Significant differences were found in concentration of these compounds between breeds and gender.

**Electronic supplementary material:**

The online version of this article (doi:10.1186/s13028-015-0144-9) contains supplementary material, which is available to authorized users.

## Findings

Analysis of free amino acids and acylcarnitines circulating in blood is a powerful diagnostic tool for metabolic illnesses and imbalances [1]; conditions that have an economic impact in the equine sector [2]. Reference ranges for these compounds have been established for obese rats, adult canines and even for plants by tandem mass spectrometry (MS/MS) [3–5] and atypical myopathies have been identified in horses (*Equus caballus*) using this technology [6]. Metabolic disorders are common in horses, and can be categorized as disorders of carbohydrate metabolism [7], lipid disorders [8], purine disorders [9], and primary disorders of electrolytic flux [10]. Diet [11] and metabolic disorders are important factors affecting amino acid and acylcarnitine concentrations in horses [12].

We hypothesized that concentrations of amino acids and acylcarnitines in the blood are different between Quarter horses (QH) and American Miniature horses (AMH). This study reports the concentrations of these compounds in peripheral blood of normal horse of both gender and establishes normal reference ranges for these breeds.

The research protocol was approved by the Institutional Committee for the Act # 2 (Approbation code FMVZ-CBA-002). A total of 100 clinically normal horses [50 QH (23 males and 27 females) and 50 AMH (15 males and 35 females)] from several stud farms and riding stables in the cities of Monterrey, Nuevo León and Cuatrociénegas, Coahuila, northeastern Mexico were selected. The horses were fed twice a day with dry forage (2.2 % of their body weight) and commercial cereals, molasses, oilseeds, vitamins and minerals (0.5 % of their body weight), and water ad libitum. The horses were used in racing, rodeos, or kept as pets. Age and weight ranges are shown in detail in Additional file 1.

Complete blood was collected 3–5 h after first food ingestion in the morning from the jugular vein using a vacutainer tube with no anticoagulant and a 21G × 1.5″ size needle; posteriorly one drop of blood was placed on each of five filter paper circles (S&S 903, Whatman, Little Chalfont, UK) using a pipette. The filter papers were then dried for 3 h at room temperature avoiding exposure to sunlight.

**Fig. 1:**
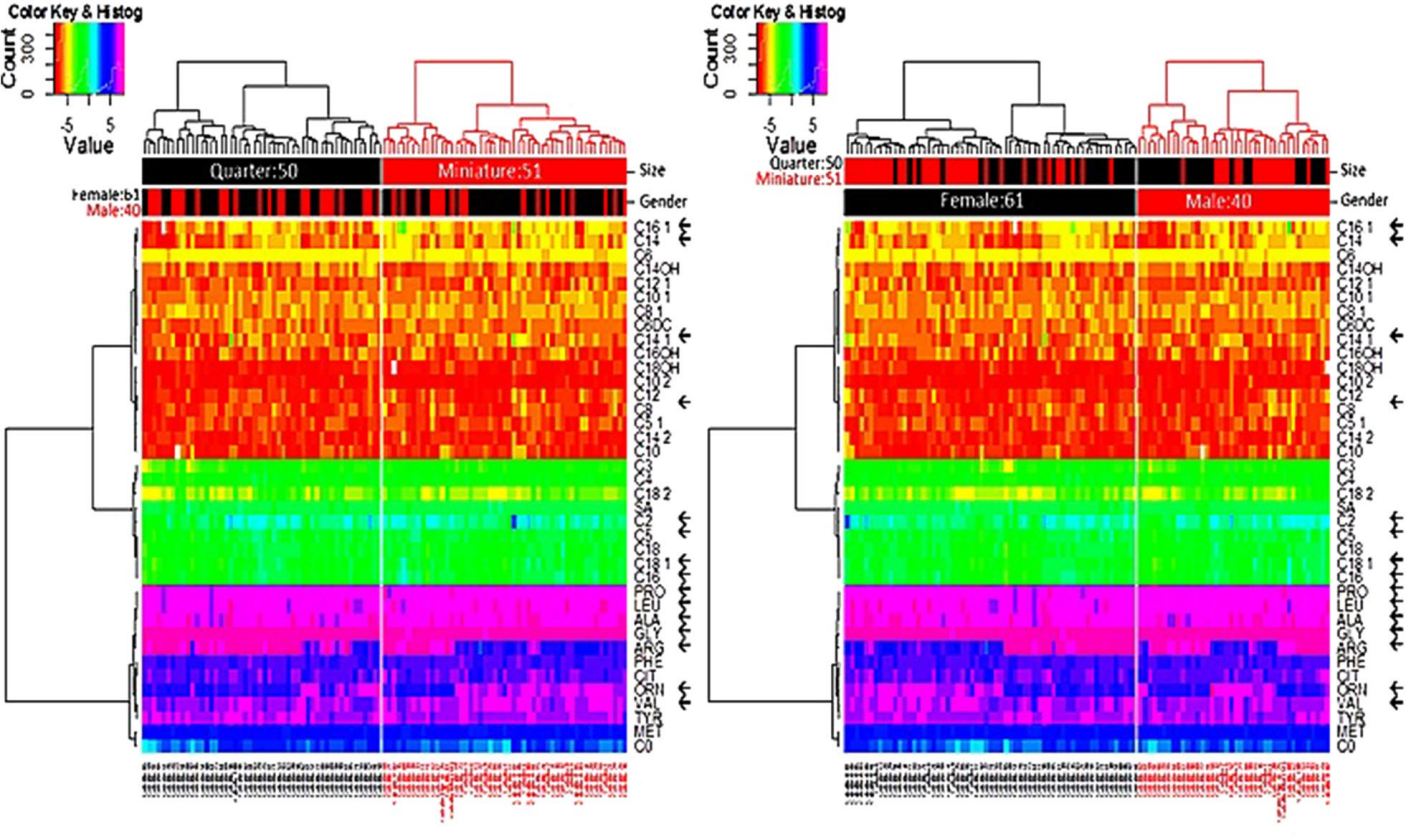
Heat map representing concentrations of metabolites analyzed. *Left panel* shows data order by size. *Right panel* shows concentrations by gender. Horses are shown in *columns* and metabolites in *rows*, which are grouped by hierarchical clustering for clarity. *Color code* is shown. *Arrows* highlight significant changes

Each sample was analyzed for 12 amino acids and 26 acylcarnitines (Fig. 1). A 3.2 mm circle was obtained using a Wallac DBS Puncher (PerkinElmer, Waltham, MA, USA) from the dry filter paper. NeoBase non-derivatized MS/MS kit (PerkinElmer) was used to obtain the metabolites of interest, following the manufacturer’s instructions. A solution included in the kit containing internal standards labeled with stable isotopes was used for quantification of the metabolites of interest. The prepared samples were analyzed by MS/MS (API 2000, ABSciex, Framingham, MA, USA) coupled to a micropump and an autosampler (Series 2000, Perkin Elmer). Sample analysis was performed with multiple reaction monitoring using Analyst 1.6.2 Software (ABSciex) and the NeoBase database. The results were interpreted using Analyst 1.6.2 Software.

Statistical analysis was performed using R (http://www.cran.r-project.org/). To normalize the raw measurements, we set the sum of all values within a sample to 1. This was achieved by dividing each value by the sum of all values per sample. These normalized values were then converted to logarithms to handle extreme values. A *t* test was used to determine statistically significant differences between groups. Corrections of q-values were made for multiple tests using a false discovery rate approach and *P* ≤ 0.05 were considered as significant. For correlation analyses, absolute Pearson correlations ≥0.7 were used.

Normal reference ranges were established for 12 amino acids and 26 acylcarnitines in blood of AMH and QH. Mean values, standard deviation, maximum and minimum values for each metabolite are provided in Additional file 2 for each breed and gender. Among the 38 metabolites (Fig. 1), the concentrations of 16 were significantly different between the horse breeds (indicated by * Table 1).

**Table 1:** Overview of analyzed metabolites in American Miniature horses (AMH) (n = 50) and Quarter horses (QH) (n = 50)

Metabolites	Compared groups			
	QH♀/QH♂	QH♀/AMH♀	QH♂/AMH♂	AMH♀/AMH♂
C0	0.87	0.44	0.73	0.93
C2	0.61	0.44	0.03*	0.86
C4	0.87	0.14	0.10	0.86
C6	0.61	0.44	0.83	0.96
C6DC	0.29	0.81	0.06	0.86
C8	0.52	0.81	0.01*	0.86
C8.1	0.99	0.07	0.35	0.86
C10	0.61	0.16	0.58	0.93
C10.1	0.80	0.45	0.14	0.86
C10.2	0.52	0.81	0.19	0.93
C12	0.36	0.81	0.06	0.86
C12.1	0.77	0.41	0.22	0.86
C14	0.80	0.06	0.01*	0.86
C14.1	0.61	0.15	0.04*	0.96
C14.2	0.61	0.81	0.14	0.86
C14OH	0.61	0.81	0.35	0.93
C16	0.80	0.15	0.01*	0.86
C16.1	0.61	0.20	0.01*	0.86
C16OH	0.90	0.98	0.74	0.86
C18	0.61	0.81	0.20	0.86
C18.1	0.61	0.29	0.01*	0.86
C18.2	0.61	0.98	0.38	0.93
C18OH	0.61	0.61	0.10	0.93
C5	0.61	0.41	0.03*	0.86
C5.1	0.61	0.41	0.12	0.86
C3	0.87	0.04*	0.03*	0.86
ALA	0.61	0.06	0.01*	0.93
ARG	0.89	0.00*	0.03*	0.86
CIT	0.87	0.45	0.07	0.86
GLY	0.36	0.45	0.03*	0.93
LEU	0.61	0.00*	0.00*	0.86
MET	0.89	0.43	0.78	0.86
ORN	0.97	0.01*	0.15	0.86
PHE	0.80	0.28	0.14	0.93
PRO	0.80	0.00*	0.01*	0.86
SA	0.61	0.23	0.22	0.86
TYR	0.61	0.11	0.38	0.86
VAL	0.87	0.00*	0.04*	0.86

The data were statistically compared according to breed (AMH vs. QH) and gender (♂ vs. ♀). See Abbreviations for metabolite abbreviations

* Represents a *P* value of *P* ≤ 0.05

Comparisons by gender between breeds (female quarters vs female miniatures, and the same for males) were statistically significant for both males and females (Table 1; Fig. 1), but not within the same breed. For male horses, concentrations of C2, C3, C5, C8, C14, C14:1, C:16 and C16:1 were significantly higher in AMH than in QH. Only C18:1 was higher in male QH than in male AMH. The concentrations of the amino acids Ala, Arg, Gly, Pro and Leu were significantly higher in QH than in AMH, while the concentrations of Val was higher in AMH than in QH. For the female horses, the concentrations of C3, Leu, Pro and Arg were significantly higher in AMH than in QH. Inversely, Val and Orn were higher in QH than in AMH (Table 1; Fig. 1).

In the present study, horse breeds showed differences in acylcarnitine concentrations, which could be caused by variation in daily physical activity, individual genetics, or environmental factors. Diet is a factor that affects blood concentrations of amino acids, hormones, proteins, and other circulating compounds [11]. In the present study, horses were fed the same ration based on their individual weight, so the effects of diet on the obtained values is considered minimal. QH showed higher Leu concentrations than AMH and conversely Val concentrations were higher in male AMH than in male QH. These differences may reflect metabolic differences between breeds. Analysis of samples from other breeds may clarify this. If the differences depend on breed, this factor should be taken into account in the interpretation of the results for the possible diagnosis of metabolic disorders.

In conclusion, normal reference ranges were established for amino acids and acylcarnitines in QH and AMH. The established ranges could be used to diagnose metabolic abnormalities that affect the performance of horses of high sporting and economic value. Positive results can be followed up with more specific testing for confirmation of the diagnosis.

## Additional files

10.1186/s13028-015-0144-9 Phenotypic characteristics of the horses of both breeds. Includes phenotypical characteristics (breed, sex, age, weight, height) and the relationship between individual of the group.
10.1186/s13028-015-0144-9 Statistic data. Includes mean, standard deviation, minimum and maximum ranges for the metabolites analyzed.

## Abbreviations

Ala: alanine
AMH: American Miniature horses
Arg: arginine
C0: free carnitine
C4: butyrylcarnitine
C5: 1: tiglylcarnitine
C6: hexanoylcarnitine
C6DC: adipylcarnitine
C8: 1: octenoylcarnitine
C10: decanoyl carnitine
C10: 1: decenoyl carnitine
C10: 2: decadienoyl carnitine
C12: dodecanoyl carnitine
C12: 1: dodecenoyl carnitine
C14: 2: tetradecadienoyl carnitine
C14OH: 3-hydroxy-tetradecanoyl carnitine
C16OH: 3 hydroxy-hexadecanoyl carnitine
C16: 1OH: 3-hydroxy-hexadecenoyl carnitine
C18: octadecanoyl carnitine (estearoyl carnitine)
C18: 2: octadecadienoyl carnitine (linoleyl carnitine)
C18OH: 3-hydroxy-octadecanoyl carnitine
C18: 1OH: 3-hydroxy-octadecenoyl carnitine
Cit: citrulline
Gly: glycine
Leu: leucine
Met: methionine
Orn: ornithine
Phe: phenylalanine
Pro: proline
QH: Quarter horses
SA: succinylacetone
Tyr: tyrosine
Val: valine
