# Supplementary material for: Reference values for amino acids and acylcarnitines in peripheral blood in Quarter horses and American Miniature horses
Source: Acta Vet Scand. 2015 Sep 29;57:62. doi: 10.1186/s13028-015-0144-9 (PMC4587867; doi:10.1186/s13028-015-0144-9)
Supplement: Supplementary file 1 — 10.1186/s13028-015-0144-9 Phenotypic characteristics of the horses of both breeds. Includes phenotypical characteristics (breed, sex, age, weight, height) and the relationship between individual of the group. [file 13028_2015_144_MOESM1_ESM.docx]

**Additional file 1 (.txt). - Phenotypic characteristics of the horses of both breeds.** Includes phenotypical characteristics (breed, sex, age, weight, height) and the relationship between individual of the group.

| **Horse ID** | **Breed** | **Sex** | **Age (Years)** | **Weight**  **(Kg)** | **Height**  **(Inches)** | **Relationship** |
| --- | --- | --- | --- | --- | --- | --- |
| **1** | AMH | ♂ | 12.16 | 110.5 | 32.5 | ND |
| **2** | AMH | ♂ | 6 | 102 | 34 | ND |
| **3** | AMH | ♂ | 7.08 | 100 | 32 | ND |
| **4** | AMH | ♂ | 5 | 103 | 32.5 | ND |
| **5** | AMH | ♂ | 16.16 | 90 | 28 | ND |
| **6** | AMH | ♀ | 6 | 100 | 34 | ND |
| **7** | AMH | ♀ | 5 | 98 | 31 | ND |
| **8** | AMH | ♀ | 5 | 99 | 34.5 | ND |
| **9** | AMH | ♀ | 6.16 | 102 | 36 | ND |
| **10** | AMH | ♀ | 0.08 | 12 | 24.5 | Foal of #9 y #3 |
| **11** | AMH | ♀ | 13.16 | 106 | 30 | ND |
| **12** | AMH | ♀ | 11.16 | 105 | 31 | ND |
| **13** | AMH | ♀ | 9.16 | 115 | 33.7 | ND |
| **14** | AMH | ♀ | 9.16 | 118 | 33 | Mother of #15 |
| **15** | AMH | ♂ | 0.25 | 16 | 21.2 | Foal of 3# y #15 |
| **16** | AMH | ♀ | 8 | 110 | 36 | ND |
| **17** | AMH | ♀ | 4.16 | 99 | 35.5 | ND |
| **18** | AMH | ♀ | 11.08 | 114 | 33.5 | ND |
| **19** | AMH | ♀ | 13.92 | 98 | 33 | Mother of #20 |
| **20** | AMH | ♂ | 0.16 | 14 | 26 | Foal of #19 y #1 |
| **21** | AMH | ♀ | 10.25 | 106 | 31.5 | ND |
| **22** | AMH | ♀ | 0.5 | 48 | 28.5 | ND |
| **23** | AMH | ♂ | 2 | 95 | 30 | ND |
| **24** | AMH | ♀ | 5.92 | 100 | 31.5 | ND |
| **25** | AMH | ♀ | 11.08 | 100 | 32.5 | ND |
| **26** | AMH | ♀ | 12.67 | 120 | 32.7 | ND |
| **27** | AMH | ♀ | 11 | 118 | 35 | ND |
| **28** | AMH | ♀ | 6.84 | 100 | 32.7 | ND |
| **29** | AMH | ♀ | 12.16 | 110 | 33.4 | ND |
| **30** | AMH | ♀ | 15 | 100 | 29 | ND |
| **31** | AMH | ♀ | 12 | 98 | 32 | ND |
| **32** | AMH | ♂ | 6 | 112 | 34 | ND |
| **33** | AMH | ♂ | 11.25 | 102 | 29 | ND |
| **34** | AMH | ♀ | 2.16 | 100 | 32 | ND |
| **35** | AMH | ♀ | 6 | 105 | 33 | Mother of #36 #43 |
| **36** | AMH | ♂ | 0.16 | 45 | 26 | Foal of #35 y #2 |
| **37** | AMH | ♀ | 7 | 105 | 35.5 | ND |
| **38** | AMH | ♀ | 6 | 104 | 35 | Mother of #39 |
| **39** | AMH | ♀ | 0.5 | 49 | 30 | Foal of #38 y #42 |
| **40** | AMH | ♂ | 6 | 100 | 32 | ND |
| **41** | AMH | ♀ | 6 | 103 | 35 | Mother of #42 |
| **42** | AMH | ♂ | 0.08 | 28 | 24 | Foal of #41 y #32 |
| **43** | AMH | ♀ | 2.33 | 101 | 32.5 | Foal of #35 y #2 |
| **44** | AMH | ♀ | 2.67 | 100 | 32.5 | Foal of #37 y #2 |
| **45** | AMH | ♂ | 3.08 | 105 | 36 | Foal of #37 |
| **46** | AMH | ♀ | 1 | 98 | 31.5 | ND |
| **47** | AMH | ♂ | 0.67 | 85 | 28.5 | Foal of #37 y #2 |
| **48** | AMH | ♀ | 2 | 100 | 30.5 | ND |
| **49** | AMH | ♀ | 5 | 110 | 33 | ND |
| **50** | AMH | ♀ | 3 | 105 | 32.5 | ND |
| **51** | QH | ♀ | 2 | 300 | 64 | ND |
| **52** | QH | ♂ | 2 | 320 | 64 | ND |
| **53** | QH | ♂ | 5 | 425 | 64.5 | ND |
| **54** | QH | ♀ | 10 | 600 | 65 | ND |
| **55** | QH | ♀ | 5 | 400 | 64.5 | ND |
| **56** | QH | ♀ | 6 | 438 | 66 | ND |
| **57** | QH | ♀ | 6 | 433 | 65 | ND |
| **58** | QH | ♂ | 11 | 602 | 66 | ND |
| **59** | QH | ♂ | 3 | 450 | 64 | ND |
| **60** | QH | ♀ | 7 | 455 | 64 | ND |
| **61** | QH | ♀ | 3 | 410 | 64.3 | ND |
| **62** | QH | ♂ | 5 | 430 | 64.2 | ND |
| **63** | QH | ♀ | 15 | 598 | 66 | ND |
| **64** | QH | ♂ | 7 | 500 | 66.1 | ND |
| **65** | QH | ♀ | 7 | 515 | 65 | ND |
| **66** | QH | ♂ | 1 | 250 | 65 | ND |
| **67** | QH | ♂ | 1 | 280 | 64.2 | ND |
| **68** | QH | ♂ | 11 | 595 | 66 | ND |
| **69** | QH | ♂ | 5 | 490 | 66 | ND |
| **70** | QH | ♀ | 5 | 480 | 64.3 | ND |
| **71** | QH | ♀ | 7 | 500 | 64.3 | ND |
| **72** | QH | ♂ | 5 | 480 | 64 | ND |
| **73** | QH | ♀ | 5 | 440 | 64.5 | ND |
| **74** | QH | ♀ | 7 | 520 | 65 | ND |
| **75** | QH | ♀ | 8 | 525 | 65 | ND |
| **76** | QH | ♀ | 5 | 445 | 66 | ND |
| **77** | QH | ♀ | 10 | 602 | 64.2 | ND |
| **78** | QH | ♂ | 1 | 400 | 64.3 | ND |
| **79** | QH | ♂ | 10 | 600 | 64.2 | ND |
| **80** | QH | ♀ | 5 | 450 | 66 | ND |
| **81** | QH | ♀ | 8 | 500 | 64 | ND |
| **82** | QH | ♀ | 10 | 603 | 64.2 | ND |
| **83** | QH | ♀ | 8 | 520 | 64.2 | ND |
| **84** | QH | ♀ | 6 | 522 | 64.5 | ND |
| **85** | QH | ♀ | 5 | 500 | 64.2 | ND |
| **86** | QH | ♀ | 7 | 532 | 65 | ND |
| **87** | QH | ♂ | 12 | 602 | 65 | ND |
| **88** | QH | ♂ | 12 | 598 | 64.5 | ND |
| **89** | QH | ♂ | 8 | 550 | 65 | ND |
| **90** | QH | ♀ | 9 | 552 | 65 | ND |
| **91** | QH | ♂ | 12 | 600 | 66 | ND |
| **92** | QH | ♀ | 6 | 489 | 65.2 | ND |
| **93** | QH | ♂ | 16 | 600 | 65.3 | ND |
| **94** | QH | ♀ | 7 | 492 | 64 | ND |
| **95** | QH | ♂ | 14 | 595 | 64.3 | ND |
| **96** | QH | ♀ | 5 | 500 | 64 | ND |
| **97** | QH | ♂ | 1 | 400 | 64.5 | ND |
| **98** | QH | ♂ | 9 | 480 | 64 | ND |
| **99** | QH | ♂ | 11 | 593 | 64.3 | ND |
| **100** | QH | ♂ | 9 | 460 | 66 | ND |

*ND: Not Determined
