# Supplementary material for: Reference values for amino acids and acylcarnitines in peripheral blood in Quarter horses and American Miniature horses
Source: Acta Vet Scand. 2015 Sep 29;57:62. doi: 10.1186/s13028-015-0144-9 (PMC4587867; doi:10.1186/s13028-015-0144-9)
Supplement: Supplementary file 2 — 10.1186/s13028-015-0144-9 Statistic data. Includes mean, standard deviation, minimum and maximum ranges for the metabolites analyzed. [file 13028_2015_144_MOESM2_ESM.docx]

**Additional file 2 (.txt). - Statistic data.** Includes mean, standard deviation, minimum and maximum ranges for the metabolites analyzed.

| **Metabolite** | **Group** | **Mean (µmol/L)** | **SD**  **(µmol/L)** | **Min**  **(µmol/L)** | **Max**  **(µmol/L)** |
| --- | --- | --- | --- | --- | --- |
| **C0** | **QH**♀ | 0.006040 | 0.002453 | 0.002221 | 0.011975 |
|  | **QH**♂ | 0.000736 | 0.000350 | 0.000183 | 0.001398 |
|  | **AMH**♀ | 0.000228 | 0.000084 | 0.000094 | 0.000458 |
|  | **AMH**♂ | 0.000025 | 0.000007 | 0.000014 | 0.000038 |
| **C2** | **QH**♀ | 0.000009 | 0.000003 | 0.000004 | 0.000018 |
|  | **QH**♂ | 0.000006 | 0.000002 | 0.000002 | 0.000011 |
|  | **AMH**♀ | 0.000014 | 0.000004 | 0.000006 | 0.000024 |
|  | **AMH**♂ | 0.000007 | 0.000004 | 0.000001 | 0.000015 |
| **C4** | **QH**♀ | 0.000012 | 0.000004 | 0.000007 | 0.000021 |
|  | **QH**♂ | 0.000004 | 0.000002 | 0.000002 | 0.000010 |
|  | **AMH**♀ | 0.000005 | 0.000002 | 0.000001 | 0.000009 |
|  | **AMH**♂ | 0.000009 | 0.000003 | 0.000003 | 0.000017 |
| **C6** | **QH**♀ | 0.000011 | 0.000007 | 0.000001 | 0.000035 |
|  | **QH**♂ | 0.000009 | 0.000005 | 0.000001 | 0.000020 |
|  | **AMH**♀ | 0.000005 | 0.000003 | 0.000001 | 0.000012 |
|  | **AMH**♂ | 0.000012 | 0.000006 | 0.000003 | 0.000027 |
| **C6DC** | **QH**♀ | 0.000341 | 0.000159 | 0.000088 | 0.000634 |
|  | **QH**♂ | 0.000013 | 0.000007 | 0.000004 | 0.000032 |
|  | **AMH**♀ | 0.000009 | 0.000004 | 0.000004 | 0.000019 |
|  | **AMH**♂ | 0.000343 | 0.000125 | 0.000137 | 0.000634 |
| **C8** | **QH**♀ | 0.000284 | 0.000140 | 0.000075 | 0.000554 |
|  | **QH**♂ | 0.000087 | 0.000042 | 0.000024 | 0.000167 |
|  | **AMH**♀ | 0.000004 | 0.000002 | 0.000001 | 0.000007 |
|  | **AMH**♂ | 0.000357 | 0.000144 | 0.000096 | 0.000598 |
| **C8.1** | **QH**♀ | 0.000006 | 0.000003 | 0.000001 | 0.000012 |
|  | **QH**♂ | 0.000133 | 0.000062 | 0.000047 | 0.000290 |
|  | **AMH**♀ | 0.104557 | 0.025796 | 0.074176 | 0.190331 |
|  | **AMH**♂ | 0.149917 | 0.075461 | 0.016506 | 0.263229 |
| **C10** | **QH**♀ | 0.040340 | 0.011135 | 0.020627 | 0.059315 |
|  | **QH**♂ | 0.306743 | 0.067739 | 0.213052 | 0.479254 |
|  | **AMH**♀ | 0.089688 | 0.016380 | 0.057581 | 0.111680 |
|  | **AMH**♂ | 0.013089 | 0.003442 | 0.007298 | 0.019741 |
| **C10.1** | **QH**♀ | 0.042918 | 0.030059 | 0.019971 | 0.130973 |
|  | **QH**♂ | 0.033107 | 0.005782 | 0.018234 | 0.044697 |
|  | **AMH**♀ | 0.090337 | 0.017838 | 0.062151 | 0.123062 |
|  | **AMH**♂ | 0.000491 | 0.000102 | 0.000379 | 0.000760 |
| **C10.2** | **QH**♀ | 0.064015 | 0.013170 | 0.036973 | 0.093552 |
|  | **QH**♂ | 0.056089 | 0.014095 | 0.030098 | 0.083976 |
|  | **AMH**♀ | 0.005512 | 0.001919 | 0.002853 | 0.009918 |
|  | **AMH**♂ | 0.001009 | 0.000358 | 0.000517 | 0.001758 |
| **C12** | **QH**♀ | 0.000271 | 0.000066 | 0.000136 | 0.000385 |
|  | **QH**♂ | 0.000026 | 0.000009 | 0.000014 | 0.000046 |
|  | **AMH**♀ | 0.000011 | 0.000003 | 0.000006 | 0.000018 |
|  | **AMH**♂ | 0.000012 | 0.000007 | 0.000004 | 0.000033 |
| **C12.1** | **QH**♀ | 0.000015 | 0.000004 | 0.000009 | 0.000022 |
|  | **QH**♂ | 0.000007 | 0.000004 | 0.000002 | 0.000021 |
|  | **AMH**♀ | 0.000014 | 0.000004 | 0.000008 | 0.000022 |
|  | **AMH**♂ | 0.000003 | 0.000001 | 0.000001 | 0.000006 |
| **C14** | **QH**♀ | 0.000009 | 0.000005 | 0.000002 | 0.000020 |
|  | **QH**♂ | 0.000010 | 0.000002 | 0.000005 | 0.000014 |
|  | **AMH**♀ | 0.000019 | 0.000009 | 0.000006 | 0.000036 |
|  | **AMH**♂ | 0.000013 | 0.000006 | 0.000006 | 0.000027 |
| **C14.1** | **QH**♀ | 0.000007 | 0.000004 | 0.000002 | 0.000017 |
|  | **QH**♂ | 0.000010 | 0.000004 | 0.000004 | 0.000017 |
|  | **AMH**♀ | 0.000581 | 0.000357 | 0.000216 | 0.001739 |
|  | **AMH**♂ | 0.000024 | 0.000017 | 0.000008 | 0.000080 |
| **C14.2** | **QH**♀ | 0.000009 | 0.000007 | 0.000003 | 0.000031 |
|  | **QH**♂ | 0.000421 | 0.000199 | 0.000216 | 0.000968 |
|  | **AMH**♀ | 0.000478 | 0.000269 | 0.000183 | 0.001331 |
|  | **AMH**♂ | 0.000070 | 0.000030 | 0.000038 | 0.000138 |
| **C14OH** | **QH**♀ | 0.000005 | 0.000002 | 0.000001 | 0.000009 |
|  | **QH**♂ | 0.000478 | 0.000139 | 0.000263 | 0.000714 |
|  | **AMH**♀ | 0.000008 | 0.000004 | 0.000003 | 0.000015 |
|  | **AMH**♂ | 0.000174 | 0.000051 | 0.000112 | 0.000297 |
| **C16** | **QH**♀ | 0.130827 | 0.028228 | 0.089592 | 0.188688 |
|  | **QH**♂ | 0.078084 | 0.058058 | 0.014981 | 0.173920 |
|  | **AMH**♀ | 0.047324 | 0.009838 | 0.029232 | 0.065769 |
|  | **AMH**♂ | 0.258574 | 0.040672 | 0.190267 | 0.321432 |
| **C16.1** | **QH**♀ | 0.121654 | 0.018618 | 0.086782 | 0.161768 |
|  | **QH**♂ | 0.012659 | 0.002976 | 0.008158 | 0.018000 |
|  | **AMH**♀ | 0.062392 | 0.035445 | 0.017403 | 0.120282 |
|  | **AMH**♂ | 0.036471 | 0.005867 | 0.025746 | 0.043954 |
| **C16OH** | **QH**♀ | 0.114918 | 0.021103 | 0.063657 | 0.160373 |
|  | **QH**♂ | 0.000538 | 0.000107 | 0.000367 | 0.000720 |
|  | **AMH**♀ | 0.059865 | 0.009844 | 0.040304 | 0.081931 |
|  | **AMH**♂ | 0.067499 | 0.014194 | 0.041439 | 0.092391 |
| **C18** | **QH**♀ | 0.006482 | 0.002846 | 0.001360 | 0.013241 |
|  | **QH**♂ | 0.000870 | 0.000444 | 0.000178 | 0.001833 |
|  | **AMH**♀ | 0.000248 | 0.000117 | 0.000076 | 0.000564 |
|  | **AMH**♂ | 0.000028 | 0.000008 | 0.000014 | 0.000044 |
| **C18.1** | **QH**♀ | 0.000011 | 0.000003 | 0.000006 | 0.000019 |
|  | **QH**♂ | 0.000008 | 0.000003 | 0.000002 | 0.000013 |
|  | **AMH**♀ | 0.000013 | 0.000003 | 0.000007 | 0.000022 |
|  | **AMH**♂ | 0.000006 | 0.000004 | 0.000002 | 0.000019 |
| **C18.2** | **QH**♀ | 0.000013 | 0.000004 | 0.000007 | 0.000020 |
|  | **QH**♂ | 0.000003 | 0.000002 | 0.000001 | 0.000008 |
|  | **AMH**♀ | 0.000007 | 0.000002 | 0.000004 | 0.000012 |
|  | **AMH**♂ | 0.000010 | 0.000004 | 0.000004 | 0.000018 |
| **C18OH** | **QH**♀ | 0.000012 | 0.000007 | 0.000002 | 0.000029 |
|  | **QH**♂ | 0.000011 | 0.000005 | 0.000004 | 0.000029 |
|  | **AMH**♀ | 0.000006 | 0.000003 | 0.000003 | 0.000014 |
|  | **AMH**♂ | 0.000011 | 0.000005 | 0.000004 | 0.000024 |
| **C5** | **QH**♀ | 0.000372 | 0.000179 | 0.000064 | 0.000882 |
|  | **QH**♂ | 0.000016 | 0.000008 | 0.000002 | 0.000038 |
|  | **AMH**♀ | 0.000010 | 0.000004 | 0.000002 | 0.000021 |
|  | **AMH**♂ | 0.000408 | 0.000163 | 0.000053 | 0.000772 |
| **C5.1** | **QH**♀ | 0.000320 | 0.000147 | 0.000079 | 0.000752 |
|  | **QH**♂ | 0.000071 | 0.000036 | 0.000027 | 0.000148 |
|  | **AMH**♀ | 0.000005 | 0.000003 | 0.000001 | 0.000013 |
|  | **AMH**♂ | 0.000416 | 0.000184 | 0.000106 | 0.000825 |
| **C3** | **QH**♀ | 0.000007 | 0.000004 | 0.000002 | 0.000015 |
|  | **QH**♂ | 0.000150 | 0.000086 | 0.000027 | 0.000392 |
|  | **AMH**♀ | 0.115120 | 0.027690 | 0.071754 | 0.174346 |
|  | **AMH**♂ | 0.161366 | 0.090596 | 0.025345 | 0.297430 |
| **ALA** | **QH**♀ | 0.041063 | 0.010714 | 0.024054 | 0.067104 |
|  | **QH**♂ | 0.268919 | 0.053714 | 0.197666 | 0.405829 |
|  | **AMH**♀ | 0.096991 | 0.025939 | 0.053938 | 0.148576 |
|  | **AMH**♂ | 0.012878 | 0.003449 | 0.006743 | 0.018941 |
| **ARG** | **QH**♀ | 0.044570 | 0.031411 | 0.018166 | 0.116609 |
|  | **QH**♂ | 0.034292 | 0.006837 | 0.018915 | 0.047875 |
|  | **AMH**♀ | 0.087978 | 0.020717 | 0.048532 | 0.137679 |
|  | **AMH**♂ | 0.000525 | 0.000123 | 0.000376 | 0.000796 |
| **CIT** | **QH**♀ | 0.069332 | 0.011422 | 0.052657 | 0.095034 |
|  | **QH**♂ | 0.057453 | 0.015546 | 0.035085 | 0.095515 |
|  | **AMH**♀ | 0.005434 | 0.002031 | 0.002539 | 0.013836 |
|  | **AMH**♂ | 0.001012 | 0.000924 | 0.000429 | 0.006013 |
| **GLY** | **QH**♀ | 0.000280 | 0.000055 | 0.000182 | 0.000395 |
|  | **QH**♂ | 0.000026 | 0.000008 | 0.000014 | 0.000045 |
|  | **AMH**♀ | 0.000011 | 0.000003 | 0.000006 | 0.000020 |
|  | **AMH**♂ | 0.000008 | 0.000003 | 0.000003 | 0.000018 |
| **LEU** | **QH**♀ | 0.000015 | 0.000003 | 0.000009 | 0.000022 |
|  | **QH**♂ | 0.000007 | 0.000004 | 0.000002 | 0.000018 |
|  | **AMH**♀ | 0.000013 | 0.000004 | 0.000008 | 0.000024 |
|  | **AMH**♂ | 0.000004 | 0.000001 | 0.000001 | 0.000008 |
| **MET** | **QH**♀ | 0.000008 | 0.000005 | 0.000003 | 0.000027 |
|  | **QH**♂ | 0.000012 | 0.000007 | 0.000003 | 0.000043 |
|  | **AMH**♀ | 0.000016 | 0.000008 | 0.000005 | 0.000035 |
|  | **AMH**♂ | 0.000014 | 0.000009 | 0.000004 | 0.000062 |
| **ORN** | **QH**♀ | 0.000006 | 0.000003 | 0.000002 | 0.000020 |
|  | **QH**♂ | 0.000011 | 0.000005 | 0.000003 | 0.000024 |
|  | **AMH**♀ | 0.000462 | 0.000182 | 0.000120 | 0.000923 |
|  | **AMH**♂ | 0.000021 | 0.000011 | 0.000004 | 0.000056 |
| **PHE** | **QH**♀ | 0.000010 | 0.000005 | 0.000004 | 0.000019 |
|  | **QH**♂ | 0.000380 | 0.000127 | 0.000084 | 0.000597 |
|  | **AMH**♀ | 0.000379 | 0.000155 | 0.000133 | 0.000648 |
|  | **AMH**♂ | 0.000068 | 0.000025 | 0.000024 | 0.000113 |
| **PRO** | **QH**♀ | 0.000005 | 0.000003 | 0.000001 | 0.000015 |
|  | **QH**♂ | 0.000451 | 0.000134 | 0.000204 | 0.000712 |
|  | **AMH**♀ | 0.000009 | 0.000004 | 0.000002 | 0.000016 |
|  | **AMH**♂ | 0.000196 | 0.000069 | 0.000101 | 0.000355 |
| **SA** | **QH**♀ | 0.133316 | 0.027553 | 0.083821 | 0.187016 |
|  | **QH**♂ | 0.065122 | 0.066885 | 0.009555 | 0.222408 |
|  | **AMH**♀ | 0.043898 | 0.012195 | 0.029522 | 0.083136 |
|  | **AMH**♂ | 0.257401 | 0.061412 | 0.109081 | 0.434769 |
| **TYR** | **QH**♀ | 0.129348 | 0.027941 | 0.086569 | 0.193429 |
|  | **QH**♂ | 0.011724 | 0.002194 | 0.008285 | 0.017895 |
|  | **AMH**♀ | 0.067717 | 0.026758 | 0.013833 | 0.115849 |
|  | **AMH**♂ | 0.036910 | 0.005984 | 0.020724 | 0.046335 |
| **VAL** | **QH**♀ | 0.107510 | 0.020855 | 0.065728 | 0.163217 |
|  | **QH**♂ | 0.000571 | 0.000109 | 0.000367 | 0.000849 |
|  | **AMH**♀ | 0.063535 | 0.007542 | 0.052006 | 0.092359 |
|  | **AMH**♂ | 0.074089 | 0.016539 | 0.047189 | 0.104254 |
